# The formation mechanism of acute dissection of blood blister-like aneurysm and its implication of endovascular treatment

**DOI:** 10.1186/s41016-021-00245-1

**Published:** 2021-06-03

**Authors:** Zhongyin Ye, Xianli Lv

**Affiliations:** 1 School of Medicine& School of Clinical Medicine, Tsinghua University, Beijing, China; 2 Neurosurgery Department, Beijing Tsinghua Changgung Hospital, School of Clinical Medicine, Tsinghua University, Litang Road 168, Beijing, 102218 China

**Keywords:** Blood blister-like aneurysm, Pathogenesis, Treatment, Endovascular

## Abstract

**Background:**

Acute mural dissection of the anterior wall of the internal carotid artery which may contribute to the development of blood blister-like aneurysms (BBLAs) was postulated, and stenting or flow diversion treatment across the soi-disant aneurysm was reported in this study.

**Methods:**

From December 2016 to December 2018, 8 patients presenting with subarachnoid hemorrhage (SAH) due to BBLA were subjected to endovascular treatment with stent-assisted coiling. Clinical outcomes were evaluated using a clinical outcome score scale.

**Results:**

Based on angiograms, pathologic change involving the supraclinoid segments of the internal carotid artery (ICA) adjacent to BBLA was found in all patients. This pathologic change meant a focal dissection of the supraclinoid segment of the ICA which constituted the pathogenesis of BBLAs. Closed-cell, open-cell, and braided stents were used in 1, 1, and 6 patients, respectively. Complete obliteration was achieved following endovascular treatment among all 8 patients harboring BBLA. One re-bleeding successive to a closed-cell stent across the aneurysmal neck was observed. Follow-up angiograms revealed stable complete exclusion of all BBLAs from the parent vessel at 3 to 8 months. All patients had a favorable clinical outcome score of 0–1.

**Conclusions:**

Acute dissection of a focal point of the intracranial vessels underlies the development of BBLAs. Open-cell and braided-cell stent-assisted coiling may constitute appropriate treatment due to good apposition against the vascular walls. Adjunctive coils may facilitate immediate complete occlusion of BBLAs.

## Background

Focal protrusions of the neurovascular arterial intima and/or media through the arterial adventitia constitute soi-disant blood blister-like aneurysms (BBLAs). BBLA constitutes a preferable designator involving the vertebral and basilar arteries, anterior communicating artery, middle cerebral artery, arterial circle of Willis, and supraclinoid segment of the internal carotid artery (ICA) in ascending order of frequency [1, 2]. The small size, a poorly defined broad-based neck, and a fragile wall constitute the features most commonly typifying BBLA. BBLA may undergo dynamic augmentation of size and conformationally reconfigure into a saccular shape when a longitudinal axis through the center of the aneurysmal neck and apices of aneurysmal domes intersects with the optic nerve [3]. BBLA occur with exceptional rarity to constitute between 0.3 and 1% and between 0.9 and 6.5% of aneurysms involving the intracranial vasculature and intracranial ICA, respectively [4]. Female gender, hypertension, younger age, and origin from the right side of the ICA may constitute demographic and pathologic properties conspiring to enhance the risk of developing BBLA [5]. The overall peri-therapeutic mortality approximated 19% and continued ignorance of mechanisms underlying pathogenesis and optimal management for BBLA. Clinical outcomes in patients harboring BBLA continues to remain dismal irrespective of whether treatment is instituted or foregone. In a recently conducted survey of the identifiable literature, institution of surgical or endovascular therapy failed to modify clinical outcomes, lesional regrowth, rates of re-bleeding, or rates of intraprocedural or postprocedural complications [6]. Innovation in novel styles of intracranial stents continues to inspire treatment of BBLA via endovascular techniques. Developing a more intimate and nuanced understanding of the mechanisms underlying the pathogenesis of soi-disant BBLA will enhance our capacity to manage and prevent re-bleeding and regrowth of these lesions to the end of maximally annihilating lesional rupture and optimizing “patient-al” neurological outcome. The rarity of BBLA encouraged our collaborative determination to conduct a review of radiograms, demographics, presenting sets of symptomatology and signs, treatment approaches, and clinical outcomes of a cohort possessing the identical pathology subjected to endovascular treatment.

## Methods

Between December 2016 and December 2018, 8 consecutive patients harboring BBLA undergoing endovascular treatment of Beijing Tsinghua Changgung Hospital of Tsinghua University were subjected to this retrospective review. The epidemiological and clinical information of these patients was summarized in Table 1. Three were men, and 5 were women. The patients’ age ranged between 37 and 60 years (mean 49 years). All patients presented with aneurysmal hemorrhage. Patients underwent computed tomography, computed tomographic angiography, and digital subtraction angiography successively. One of 8 patients had an overzealous intake of alcohol-spiked beverages. Cephalgia constituted the chief complaint in 7 patients and deep stupor in 1 patient. All patients underwent neurological examination, diagnosis, interval management, and definitive preliminary exclusion of the lesion from the intracranial circulation at a time not exceeding 72 h successive to the official admission to our infirmary.

**Table 1:** Summary of 8 BBLAs

Age/sex	H-H grade	Medications	Stents	Immediate results	Complications	Follow-up	Clinical outcome score
50/M	I	Dual antiplatelets	Enterprise	Com	Regrowth, re-bleeding	6 mon	1
56/M	I	Dual antiplatelets	Neuroform EZ	Com	Vessel spasm	6 mon	0
37/F	IV	Tirofiban	LVIS	Com	Hydrocephalus	8 mon	1
60/F	I	Dual antiplatelets	LVIS	Com	No	3 mon	0
48/F	I	Dual antiplatelets	LVIS	Com	No	6 mon	0
46/F	III	Dual antiplatelets	LVIS	Com	No	3 mon	0
48/F	I	Dual antiplatelets	Pipeline	Com	No	3 mon	0
51/M	III	Dual antiplatelets	Pipeline	Com	Vessel spasm	6 mon	1

*M*, male; *F*, female; *H-H grade*, Hunt-Hess grade; *LVIS*, low-profile visualized intraluminal support; *Com*, complete occlusion; *mon*, months

### Endovascular treatment

**Fig. 1:**
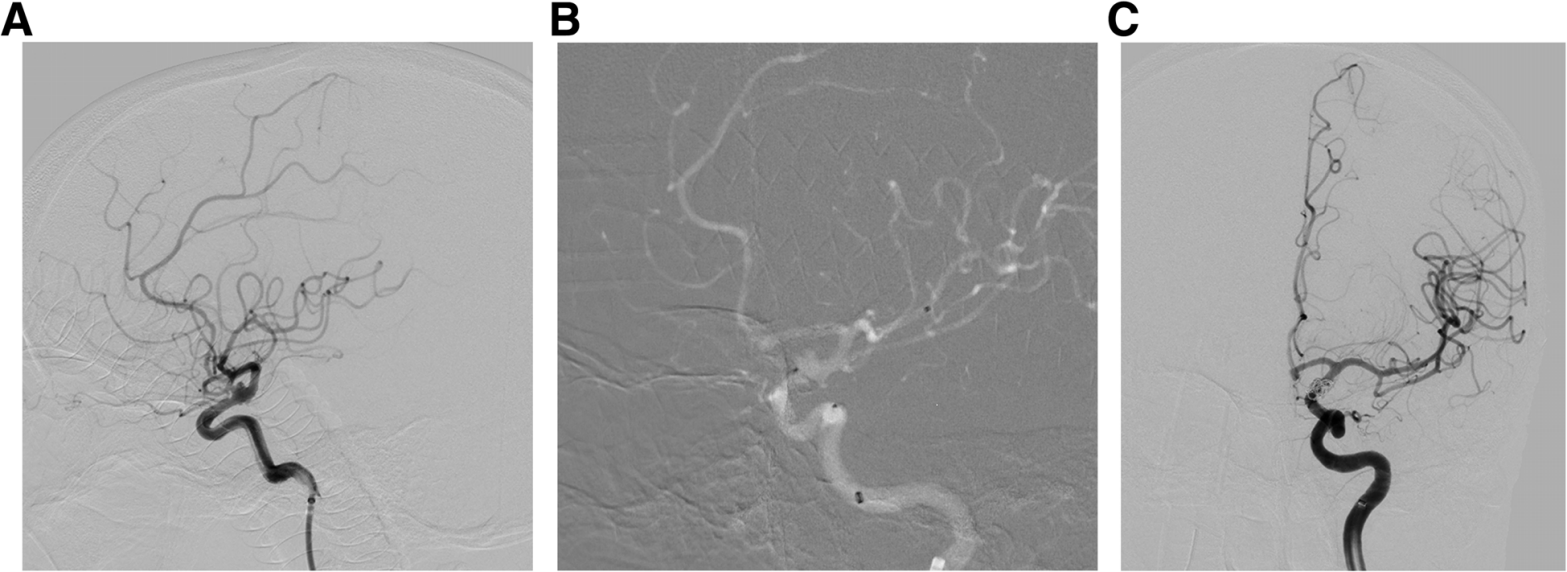
**a** Unsubtracted left ICA injection showing a broad-based BBLA of the supraclinoid ICA. **b** Under road mapping, a microcatheter was successively placed in proximity to the BBLA and deploy a 0.027-in. microcatheter across the entry point of the BBLA. **c** left ICA injection after Pipeline flow-diverting stent-assisted coiling showing the BBLA was embolized

All patients were subjected to general anesthesia. A 6-Fr guiding catheter (Envoy, Codman, Raynham, MA, USA) was selectively navigated to the ICA ipsilateral to the suspected location of the BBLA. Digital subtraction angiography (DSA) of the intracranial vasculature was achieved, and 3 dimensions permitted precise determinations of parent arterial diameter and aneurysm fundal sizes. We intended to successively place a microcatheter (Echelon 10, Medtronic, Irvine, CA, USA) in proximity to the intracranial aneurysm and deploy a stent (Enterprise, Codman, Raynham, MA, USA; Neuroform EZ, Stryker, Bosten, USA; LVIS, Microvention, Aliso Viejo, USA) across the entry point into the BBLA to the effect of jailing the preceding placed microcatheter. A few patients underwent successive sequential placement of a Pipeline flow-diverting stent (Medtronic, Irvine, CA, USA) across the internal ostium of the BBLA via a tri-axial guiding catheter including an 8-Fr guiding catheter (Envoy, Cordis, Raynham, MA, USA), a 6-Fr intermediate catheter (Navien, Medtronic, Irvine, CA, USA), and a 0.027-in. microcatheter (Marksman, Medtronic, Irvine, CA, USA) and adjunctive coiling. Coils were negotiated to approach close proximity with the aneurysmal neck, and the stent was subsequently placed to constrain the coils within the aneurysmal fundus (Fig. 1).

### Medications

The patient will be successively treated with 300 mg of aspirin and 300 mg of clopidogrel via the oral route or via nasogastric tube 3 h in precession to stent deployment across the ostium of the BBLA. Successive preprocedural bolus (0.005 mg/kg, 4–5 mL 5%) and continuous postprocedural infusions of the glycoprotein IIb/IIIa antagonist tirofiban were employed to reduce cross-linking of platelets via fibrinogen monomers. Dual synergistic therapy with 100 mg of aspirin and 75 mg of clopidogrel was administered to patients via the oral route 3 months successive to treatment, with aspirin monotherapy continued 6 months successively.

### Clinical outcome evaluation

Clinical outcome scores commensurate with 0, absence of any neurological dysfunction compromising daily functioning; 1, mild reduction of neurological function causing mild deficits in daily functioning; 2, moderate reduction of neurological function causing moderate deficits in daily functioning; 3, severe reduction of neurological function causing severe deficits in daily functioning; and 4, death.

## Results

All BBLAs among our patients involved the supraclinoid segments of ICA and were attended by luminal change. The average height of the aneurysms determined by 3-dimensional reconstructions of DSA was 3mm, ranging between 1.4 and 5.7mm. One patient was treated with 1 closed-cell stent (Enterprise), 1 patient with 1 open-cell stent (Neuroform EZ), and 6 patients with braided stents (4 LVIS and 2 Pipeline flow-diverting). Coils were successively placed to occlude BBLA in all patients achieving immediate complete obliteration by DSA.

One patient experienced postoperative re-bleeding successive to the placement of a closed-cell stent across the ostium of the BBLA. One patient presented with non-occult hydrostatic hydrocephalus. No patient developed clinically significant large vessel vasospasm. A continuous cerebrospinal fluid diversion was performed in the sole patient presenting with hydrostatic hydrocephalus. Follow-up angiography conducted 3 to 8 months successive to treatment demonstrated complete aneurysmal obliteration in all patients. Neurological function at 3 and 8 months of follow-up was classified into scores of 0 and 1, respectively.

## Discussion

Though BBLAs involve other segments of the arteries irrigating the contents of the supratentorial or infratentorial cavities within the intracranium, the supraclinoid extent of the intracranial ICA constitutes the most classic location [6]. CTA and DSA are the best efforts of veteran investigators in selecting the most appropriate therapeutic modality for BBLA [4, 5]. Endovascular reconstruction of the segment of intracranial ICA containing the ostium of BBLA achieves the end of lesional obliteration in all patients.

Subintimal dissection extending from the supraclinoid extent of the intracranial ICA through variable extents into the middle cerebral artery likely underlies the development of BBLA. Guo and colleagues [7], Yanaka and colleagues [8], and Horie and colleagues [9] identified a transmural tear identified by visual inspection intraoperatively, and an intramural hematoma was identified by dedicated magnetic resonance imaging sequences preoperatively [8]. The presence of these sub-pathognomonic hallmarks typifying soi-disant pseudoaneurysms strongly corroborated the hypothesis that focal dissection underlies the development of BBLA, the precise elucidation of which will permit the optimization of treatment paradigms.

Hemodynamic shear stress impinges upon the curvilinearly contoured anterior wall of the ICA may reduce the threshold of a traumatic event between the fixed intracavernous and mobilize the supraclinoid segments of the ICA [10]. Spiral extension and weakening of the vessel wall of the supraclinoid extent of ICA facilitate focal enlargement of the vessel at zones of separation of the layers constituting the vessel wall. Laplace’s relationship states intraluminal pressure (*P*) exerted by a column of fluid within an expandable vessel exhibits direct and inverse proportionality with 2 times of the vessel wall tension (*T*) and vessel radius (*r*) (i.e., 2*T*=*P*×*r*). Relative flattening of the supraclinoid extent of the ICA from a contoured bulk of the cavernous segment reduces ICA radius to the end reducing ICA wall tension.

Cerebral ischemia constituted a presenting symptom in 73% of patients with dissections involving the middle and anterior cerebral arteries, anterior communicating artery, and bifurcation of the ICA. The prevalence of subarachnoid hemorrhage (SAH) successive to dissection of the ICA was reported to range between 12 and 20% among afflicted patients [11]. Contraverting SAH reportedly constitutes the major manifesting presentation in upwards of 99% of patients successively identified to harbor a BBLA. “String,” an extremely thin and meandering column of contrast interposed between two apposing segments of the vessel otherwise exhibiting normal caliber, or “pearl and string,” an extremely thin and meandering column of contrast interposed between two apposing segments of vessel otherwise exhibiting normal caliber adjacent to a small micro-puddle-like collection of contrast on DSA typifying stable chronic separation of the layers constituting the vessel wall, indicates the dissection commences within the micro-slab of extracellular matrix separating the internal elastic lamina from the irregularly stratified layer of vascular smooth muscle cells [12–14].

The separative cleavage plane immediately preceding instances of SAH typically propagates from the extracranial to the intracranial extents and expansion between the tunica media and tunica adventitia [15]. Dynamic systolic arterial blood pressure, site of vessel involvement, geometry of vessel curvature, and pattern of side branches dictate successive dynamic patterns of multivariate propagation of vessel wall separation and rates of hemorrhage. Dynamic separation of the layers of the vessel wall propagating dynamically and multivariately from a focal point indicates endovascular reconstruction may constitute an effective modality excluding the false aneurysmal fundus from the parent vessels. Iatrogenic intraoperative bleeding occurs among 20% of patients successive to trephination, and microsurgical treatment of these lesions frequently causes disfavorable patient neurological outcomes [16]. Successive sequential Sylvian dissection and distoproximal peri-arterial separation of arachnoid fibers are cumbersome to faithfully conduct in the presence of edematous lobar opercula in patients developing Hunt-Hess high-grade SAH [17, 18]. Endosaccular coil embolization of fundi aneurysm oft fails to prevent instances of re-bleeding from or regrowth of BBLA [19]. Endovascular preclusion of blood entry into a segment of the intracranial extent of the ICA is perverted by an aneurysmal ostium effectively excluding BBLA from traveling columns of blood under high pressure [17, 18]. Surgically or endovascularly, occlusion of the lumen of the ICA may render insufficient to provide blood supply supporting microcirculatory perfusion flow from the contralateral ICA to cause disfavorable neurological outcomes of patients.

Wild-type opacification of the middle and anterior cerebral arteries is through the anterior communicating artery successive to bolus injections of iodinated contrast within the contralateral ICA. But the anterior and posterior communicating artery calibers usually fail to exceed or recede greater than two standard deviations from the mean of Gaussian- or non-Gaussian-distributed vessel calibers. The proximally related large vessel vasospasm and high intracranial pressure putatively can also attenuate dynamic rates of flow through the cerebral collaterals [20]. Re-establishing flow bypassly to the vessels of the intracranium irrigates the neural substance from extracranial conduits, and endovascular occlusion of segments of aneurysmal ostium may effectively attenuate the risk of BBLA rupture [18]. The prevalence of neurologic sequelae of a thromboembolic and/or hemorrhagic etiology can not be irrivisable. Therapies preserving blood flow of the ICA yield a benign clinical course. The prevalence of morbidity and death ensuant from expeditions seeking to exclude BBLA from the intracranial vasculature microsurgically or endovascularly approximated 20% and 10.7% and 7.0 and 9.0%, respectively, in a survey retrospectively conducted upon a systematic review of 334 patients [21]. A specific type of endovascular technique and treatment instituted to exclude a BBLA from the intracranial circulation dictates the obliteration rates and neurological outcomes. Therapy of employing aneurysmal coiling, placement of flow-diverting stent across the ostium of BBLA, stent-assisted coiling, or stents across the ostium of BBLA actualizes good outcome in 52.9%, 82.2%, 85.2%, and 86.4% of patients, respectively. Immediate and delayed interval (mean 20.9 months) rates of obliteration of BBLA approximated 88.9% and 88.4% in the surgical group and 63.9% and 75.9% in the endovascular group in a systematic survey of 36 peer-reviewed articles, respectively, detailing the experiences of 256 patients during 2005 through 2015 [22]. The rates of intra- and post-procedural complications in patients undergoing microsurgical and endovascular treatment were 27.8% (95%CI, 19.6–37.8%) and 26.2% (95%CI, 18.4–35.8%), respectively.

The authors have attempted to place closed-cell stents across the ostium or overlapping stents with or without packing the aneurysm with coils to exclude BBLA. The placement of open-cell stents across the ostia of BBLA endovascularly reconstructing the lumen of involved parents vessels achieves acceptable rates of stable lesional obliteration according to the description of Fiorella and colleagues [23]. Placement of closed-cell stents across the ostia of BBLA was putatively culprit in the successive development of aneurysmal regrowth and lesional hemorrhagic re-rupture in 2 and 7 patients, respectively [24]. One of our patients developing re-bleeding from the BBLA underwent placement of a closed-cell stent across the ostium in our earliest experience [13]. Incomplete apposition between closed-cell stent and segments of the vessel of BBLA enhances the risk of sequential lesional re-rupture after the procedural intervention. Stenting across the ostia of BBLA and coiling of the aneurysm constituted typical endovascular treatment at our infirmary. Placement of novel LVIS and Pipeline flow-diverting stents with a braided-cell design exhibiting dense porosity (smaller holes in the wall of the stent compared with precedingly employed closed-cell stents) across the ostia of BBLA proved safe and effective in our experience [25, 26]. Feasibility of surgical or endovascular reconstruction of segments of the parent vessels of BBLA appears to constitute a method preferentially recapitulating physiological flow through the ICA.

In a recent review conducted by Szmuda and colleagues, 56 individuals amalgamated from 17 peer-reviewed manuscripts harboring BBLA involving the supraclinoid segment of the intracranial ICA excluded by the placement of flow-diverting stents across the ostia retrospectively evaluated from 2010 to 2016 [6]. Thirty-four patients were treated using a single flow-diverting stent, 17 underwent placement of 2 flow-diverting stents and placement of single flow-diverting stent, and occlusion of the lesion with coils in 3 patients to exclude BBLA. The immediate complete obliteration rates were 27.3%, 18.8%, and 100% by the 3 strategies, respectively. Patients unfortunately returning to secure lesion obliterate in 5, among whom 3 patients died. The Modified Rankin Scores of 0–2 were in 83.3% of these patients. Persistent flow to opacify aneurysmal pseudo-fundi after placement of stents across the ostia of BBLA leading to unfortunate instances of re-rupture and premature untimely expiration underscores deficiencies in currently extant therapies. The presumptive hastening of complete exclusion by the concomitant placement of coils to occupy aneurysmal fundi validates the demonstration by previous authors that the strategy may constitute putatively durable strategies to treat BBLA.

None of our patients experienced re-rupture after placement of flow-diverting stents across the ostia of BBLA. Requisiteness of dual antiplatelet therapy maximally diminishes the risk of hemorrhagic morbidity complicating the post-procedural course of endovascular reconstruction to exclude BBLA. Development of hydrostatic hydrocephalus secondary to SAH requiring diversion of the cerebrospinal fluid from the cavities of the ventricles yields bleeding between 3 and 10% of individuals receiving antiplatelet therapy precedingly undergoing stent-assisted coiling according to a study by Bodily and colleagues [5, 27].

### Limitations of our study

Retrospective evaluation of the experience of a single institution, paucity of sampled patients, cohort heterogeneity, short span of follow-up, and/or possible selection bias and/or reporting bias constitutes the chief features of reducing the extendability of our results to refute a non-“a priori”-determined null hypothesis indicating parity between non-treatment, placebo, and treatment in acute dissection.

## Conclusions

BBLAs constitute acute separation of the layers of the vessel wall. BBLAs can be excluded from the intracranial circulation via the placement of open-cell and braided-stents across the ostia and placement of coils to occupy aneurysmal fundi facilitating their immediate complete obliteration.

## Data Availability

Please contact the authors for data requests.

## Abbreviations

BBLA: Blood blister-like aneurysm
SAH: Subarachnoid hemorrhage
ICA: Internal carotid artery
CTA: Computed tomography angiography
DSA: Digital subtraction angiography
